# Emerging Therapeutic Strategies in Sarcopenia: An Updated Review on Pathogenesis and Treatment Advances

**DOI:** 10.3390/ijms25084300

**Published:** 2024-04-12

**Authors:** Alfred Najm, Adelina-Gabriela Niculescu, Alexandru Mihai Grumezescu, Mircea Beuran

**Affiliations:** 1 Department of Surgery, Carol Davila University of Medicine and Pharmacy, 8 Eroii Sanitari, Sector 5, 050474 Bucharest, Romania; alfred.najm@yahoo.ro (A.N.); drmirceabeuran@yahoo.com (M.B.); 2 Emergency Hospital Floreasca Bucharest, 8 Calea Floresca, Sector 1, 014461 Bucharest, Romania; 3 Research Institute of the University of Bucharest—ICUB, University of Bucharest, 050657 Bucharest, Romania; adelina.niculescu@upb.ro; 4 Department of Science and Engineering of Oxide Materials and Nanomaterials, National University of Science and Technology Politehnica Bucharest, 011061 Bucharest, Romania

**Keywords:** sarcopenia pathophysiology, sarcopenia treatment, new therapeutic agents, stem cell therapy, drug delivery, tissue engineering, clinical trials

## Abstract

Sarcopenia is a prevalent degenerative skeletal muscle condition in the elderly population, posing a tremendous burden on diseased individuals and healthcare systems worldwide. Conventionally, sarcopenia is currently managed through nutritional interventions, physical therapy, and lifestyle modification, with no pharmaceutical agents being approved for specific use in this disease. As the pathogenesis of sarcopenia is still poorly understood and there is no treatment recognized as universally effective, recent research efforts have been directed at better comprehending this illness and diversifying treatment strategies. In this respect, this paper overviews the new advances in sarcopenia treatment in correlation with its underlying mechanisms. Specifically, this review creates an updated framework for sarcopenia, describing its etiology, pathogenesis, risk factors, and conventional treatments, further discussing emerging therapeutic approaches like new drug formulations, drug delivery systems, stem cell therapies, and tissue-engineered scaffolds in more detail.

## 1. Introduction

Skeletal muscle represents the most abundant tissue of the human body, accounting for 40% of the overall weight. A highly specialized tissue, skeletal muscle is involved in numerous dynamic functions (e.g., locomotion, posture control, force generation, mastication, and respiration) and metabolic processes (e.g., substrate storage for other tissues and heat generation) [1,2,3]. Given the significant rise in the aging population worldwide and the fact that muscular mass and function deteriorate with age, skeletal muscle issues have become more widespread. Addressing and managing age-related chronic conditions, such as obesity, sarcopenia, and osteoporosis, has become increasingly urgent due to their negative impact on quality of life [4,5,6,7].

Notably, sarcopenia significantly burdens society and the healthcare system, being characterized by muscle mass and function decline with age, resulting in diminished mobility, restrained posture maintenance, and reduced overall well-being [4,6,7]. With an estimated prevalence of 10–16% of the global elderly population, 18% of diabetics, and 66% of patients with unresectable esophageal malignancies, sarcopenia is associated with an elevated risk of various negative health outcomes, counting reduced overall and disease-progression-free survival rates, complications after surgery, extended hospital stays in patients with different medical conditions, as well as increased likelihood of falls, fractures, metabolic disorders, cognitive impairment, and mortality in the general population [8,9].

This degenerative skeletal muscle condition affects muscle strength, quantity, and quality of older individuals, being generated by a series of complex interrelated internal (e.g., inflammation, autophagy, mitochondrial dysfunction, defective myogenesis) and external factors (e.g., poor diet, lack of physical activity, hormone imbalances) [4,6,10,11,12]. Given the intricate nature of sarcopenia, understanding the various risk factors and recognizing the significance of preventive and control techniques are critical stages in managing this burdening disease.

In this context, this review aims to create an up-to-date framework for sarcopenia, starting with its etiology, pathogenesis, risk factors, and conventional treatment approaches and moving to emerging therapeutic strategies. Specifically, this paper overviews the recent developments concerning novel synthetic and natural therapeutic formulations, drug delivery methods, stem cell therapies, and tissue-engineering scaffolds that hold promise for future implementation in sarcopenia treatment. Moreover, completed interventional studies on sarcopenia are reviewed to offer a complete and updated perspective of the current research status against this disease.

## 2. Sarcopenia—Etiology, Pathogenesis, and Risk Factors

Sarcopenia is a comprehensive degenerative skeletal muscle disease usually affecting the elderly population. It encompasses a pathological reduction in muscular strength, mass, and function [10,11]. Two types of sarcopenia can be distinguished: primary and secondary. The primary disease is age-related, while secondary sarcopenia has been linked with various causes, including decreased physical activity levels, preexisting health issues, and poor nutrition [13].

**Figure 1:**
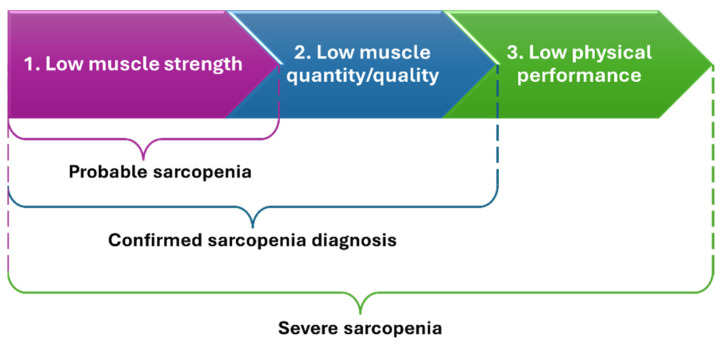
2018 operational definition of sarcopenia. Created based on information from [14,15].

The concept of sarcopenia remains a topic of debate, with no widely acknowledged consensus on its diagnosis [14]. The diagnostic criteria for sarcopenia rely on examining muscular strength, mass, and performance [10]. Sarcopenia is classified as severe when low muscular strength, quantity/quality, and physical performance are registered together [14,15] (Figure 1). Possible tools for diagnosis include dual-energy X-ray absorptiometry, bioelectrical impedance analysis, computed tomography, magnetic resonance imaging, gait speed assessment, and muscle biopsy [15].

While the exact cause is unknown, research indicates a complex interaction of intrinsic and extrinsic variables. In skeletal muscle, dysregulation in processes such as inflammation, apoptosis, autophagy, mitochondrial dysfunction, neuromuscular junction degradation, and disturbed calcium metabolism leads to defective myogenesis and muscle atrophy. Extrinsic factors such as hormone imbalances, poor diet, immobilization, and systemic inflammation aggravate muscle deterioration [4,12].

Furthermore, age is an essential factor in sarcopenia onset, with inadequate physical activity and altered gene regulation being two critical processes driving its advancement [4,12]. Compared with other age-related illnesses that facilitate sarcopenia (e.g., neurodegenerative disorders, cardiovascular diseases, respiratory conditions, diabetes mellitus, and osteoporosis), in this disease, the homeostasis of muscle mass and strength is preserved by balancing hormonal and nutritional factors with physical exercises [6]. Additionally, aging-related systemic inflammation, characterized by increased proinflammatory cytokines (e.g., IL-1β, IL-6, and TNFα) and reduced levels of anti-inflammatory cytokines, exacerbates muscle tissue microenvironment changes, impairing muscle regeneration. This chronic low-grade proinflammatory state, known as inflammaging, disrupts immune regulation and contributes significantly to the progression of sarcopenia [5,16]. Moreover, high levels of 15-hydroxyprostaglandin dehydrogenase (15-PGDH), the enzyme responsible for degrading prostaglandin E2 (PGE2), have been identified as a hallmark of aged skeletal muscle tissue [4]. This age-related decline in muscle quality is exacerbated by factors such as reduced protein intake, impaired anabolic pathways mediated by growth hormone (GH)/insulin-like growth factor (IGF) and vitamin D, and chronic low-grade inflammation, all of which contribute to disruptions in muscle bioenergetics, particularly mitochondrial metabolism [6]. Mitochondrial dysfunction has been indicated as a central pillar of muscle atrophy, with alterations in biogenesis, morphology, function, and dynamics playing key roles in disrupted muscle function and quality [4,6].

Additionally, sarcopenia is intricately linked to the progressive decline in the regenerative capacity of skeletal muscle stem cells (satellite cells, or SCs) [4,7,17]. Changes in the SC niche [18] and declines in intrinsic SC function [19] occurring with aging further contribute to impaired muscle regeneration. The age-related alterations in the extracellular matrix (ECM) of muscle tissue, characterized by increased stiffness due to the accumulation of advanced glycation end products and collagen, negatively impact SC numbers and function, exacerbating sarcopenia progression. Ultimately, these processes lead to chronic fibrosis within the muscle, further impairing regeneration and exacerbating the progression of sarcopenia [4,18,19,20,21].

An important role in satellite cells’ regenerative capacity is played by autophagy. Specifically, autophagy maintains satellite cells in a quiescent state (through the inhibition of cellular senescence and stemness), facilitates their activation in the majority of cases, accelerates the proliferation of young satellite cells, regulates mitochondrial function, mitigates basal inflammation, and promotes ROS detoxification. Thus, in the absence of autophagy-regulated regenerative ability of satellite cells, sarcopenia and fibrosis can occur and progress [22].

Additionally, sarcopenia has been associated with several underlying conditions, such as reduced secretion of testosterone, growth hormone, and ghrelin, abnormal myokine production, decreased muscle protein synthesis, and/or increased skeletal muscle protein breakdown [13]. The pathophysiology of sarcopenia has also been linked to various interrelated mechanisms related to the preservation of motor neurons, motor unit remodeling, impaired neuromuscular junction integrity, defects in excitation–contraction coupling, and potential alterations in metabolism [23].

Other risk factors for developing sarcopenia include obesity, malnutrition, inactivity, early growth environment, and comorbidities [23,24]. Conditions such as chronic kidney disease (CKD) and the necessity for dialysis introduce a cascade of factors exacerbating sarcopenia risk, including inflammation, undernutrition, and hypoalbuminemia, leading to protein-energy wasting. In more detail, dialysis causes inflammation, which leads to higher levels of acute-phase reactants such as albumin, fibrinogen, and C-reactive protein. Undernutrition exacerbates this by depleting amino acids, losing them to dialysate, and generating acute-phase proteins, which impedes muscle protein synthesis. Hypoalbuminemia exacerbates the condition, causing the body to catabolize muscle and tissue protein, releasing amino acids to keep plasma levels stable. Muscles respond by releasing local cytokines, which feed a vicious cycle of inflammation and catabolism. Chronic renal illness and dialysis frequently result in secondary sarcopenia, which indicates protein-energy loss. While protein and amino acid intake might address sarcopenia, excessive consumption may injure the kidneys, increasing disease progression through increased intraglomerular pressure [14].

On the other hand, obesity has been associated with immune aging [16]. Moreover, the condition of sarcopenic obesity implies reduced lean body mass and excess adipose tissue, mostly encountered in older people. Obesity complicates sarcopenia, promoting fat insertion into muscle, reducing endurance, and raising mortality risk [15]. Sarcopenia and obesity have a complex interaction of pathophysiological mechanisms (e.g., insulin resistance, hormonal changes, increased proinflammatory cytokines, and the presence of oxidative stress), these diseases potentiating each other [25]. Additionally, as both sarcopenia and obesity are linked to metabolic disorders, sarcopenic obesity may have a more prominent influence on metabolic diseases and cardiovascular disease-associated mortality than each condition alone [26,27]. Sarcopenic obesity has also been associated with higher insulin resistance [28], increased risk of mobility disability [29], enhanced risk of postoperative infections following cardiac surgery [30], and adverse clinical outcomes in patients who have severe Crohn’s disease [31]. Moreover, sarcopenic obesity is common in cancer patients and increases the risk of dose-limiting toxicity, surgical complications, lower functional status, and shorter survival [32].

Furthermore, the presence of cancer compounds the risk and accelerates muscle aging through mechanisms such as epigenetic changes, increased somatic mutation frequencies, muscle stem cell dysfunction, and inflammation [10]. Disease-related sarcopenia can also be caused by cachexia, a complex metabolic syndrome also found in cancer patients, that leads to a negative protein–energy balance, anorexia, and metabolic abnormalities [13,33,34,35]. On top of that, sarcopenia is linked to poor survival in oncological patients. Sarcopenia in gastric cancer patients has been linked to mortality, surgical complications, high hospital expenses, and prolonged postoperative hospital stay. Patients with sarcopenia have lower overall and cancer-specific survival rates in renal cell carcinoma and urogenital malignancies than those without sarcopenia. Sarcopenia has also been reported to reduce overall and recurrence-free survival rates in head and neck cancer patients [13,36,37].

Secondary sarcopenia may occur as a result of physical inactivity caused by prolonged bed rest, weightlessness, and anorexia, situations often encountered in hospital-associated deconditioning and disuse muscle atrophy. Disease occurrence is also favored in cases of undernutrition when there is inappropriate protein consumption, and the energy intake is significantly lower than the energy spent by the organism [13]. The consumption of protein in one’s diet is crucial for preserving muscle mass in elderly individuals. This is because amino acids, such as leucine, stimulate the mTORC1 pathway through the Rag guanosine triphosphatase (Rag GTPase) mechanism. Malnutrition worsens the imbalance between muscle protein synthesis (MPS) and muscle-protein breakdown (MPB), leading to a higher risk of sarcopenia in individuals over the age of 65. This risk can be reduced by consuming high amounts of protein and vitamin D. Regular exercise plays a crucial role in activating the mTORC1 pathway, while being inactive increases the likelihood of developing sarcopenia. Even a short period of inactivity, as little as 2 days, can significantly decrease muscle volume. Additionally, increasing sedentary behavior by 1 h per day raises the possibility of developing sarcopenia by 1.06 times [24].

Sarcopenia also exacerbates the physical functionality and prognosis of people suffering from various other health issues, including cardiovascular diseases, inflammatory bowel disease, cirrhosis, hip fractures, and surgical repair of abdominal aortic aneurysms [13,38]. Besides physical problems, sarcopenia is connected to cognitive impairment and mental decline, being also associated with depression [39,40,41].

**Figure 2:**
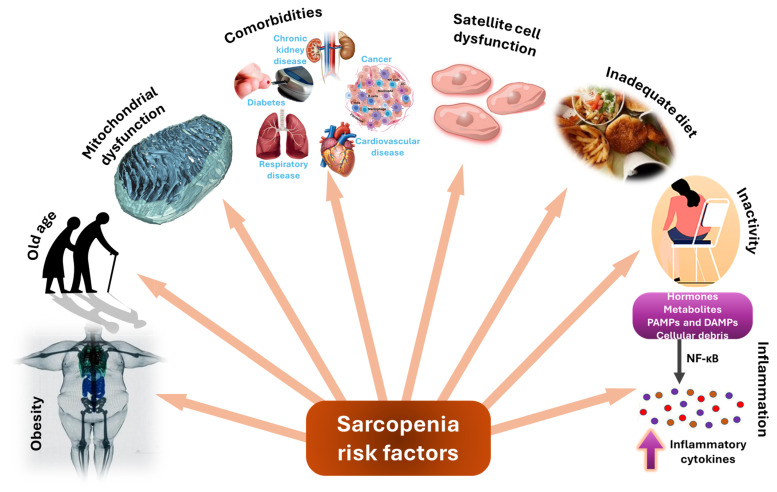
Overview of sarcopenia risk factors.

With such a complex background (Figure 2), understanding the diverse risk factors and emphasizing the importance of comprehensive preventive and control strategies are important steps in managing sarcopenia.

## 3. Conventional Treatment Approaches

While there is no treatment recognized as universally effective against sarcopenia, several strategies are currently employed in practice, generally combining exercise therapy and nutritional approaches. The choice between potential treatment options depends on patients’ characteristics, such as age, disease severity, inflammation levels, and comorbidities [13,42,43,44]. Thus, a personalized plan has to be created for each patient.

Physical activity and nutritional status represent key regulators of muscle metabolism and phenotype, reflecting their utility for accomplishing healthy aging [23]. Lifestyle modifications are essential for ensuring better autonomy for older people. Specifically, physical exercises, such as stretching exercises, coordination exercises, and resistance training, have been demonstrated effective in improving muscle strength and function, subsequently enhancing flexibility and balance [12,23,43,44].

Resistance exercise training is currently approached as a first-line treatment for sarcopenia, being a superior form of physical activity for enhancing muscle mass and strength [45,46]. Resistance exercise is considered a promising intervention, yet appropriate prescription is necessary for maximizing desirable effects. In addition, there is limited availability of relevant clinical trials, necessitating more in-depth studies for establishing full evidence of resistance training utility in older adults diagnosed with sarcopenia [46,47].

On the other hand, endurance training excels in maintaining and improving maximum aerobic power [45]. Such exercises stimulate ATP production in mitochondria within skeletal muscle, increase aerobic capacity, and improve metabolic regulation and cardiovascular function. Moreover, aerobic exercise contributes to several processes, such as induction of mitochondrial biogenesis and dynamics, mitochondrial metabolism restoration, reduction in catabolic gene expressions, and enhancement of muscle protein synthesis [48].

Adding to physical activity, a balanced diet is another important aspect of maintaining muscle mass and function. Compared to healthy subjects, sarcopenia patients were noted to consume lower amounts of certain macronutrients, such as lipids and proteins, and micronutrients, like iron, phosphorus, magnesium, potassium, and vitamin K [42]. In the elderly population, adequate protein intake is a challenging aspect, being dependent on different variables, such as anorexia of aging, anabolic resistance, molecular content, and intake patterns throughout the day [49]. Thus, for sarcopenia patients, protein supplementation or a protein-rich diet can be recommended. In clinical practice, the intake of high-quality protein, amino acids (e.g., leucine and L-carnitine), or oral nutritional supplementation containing beta-hydroxy-beta-methylbutyrate is considered [50]. It was also noted that sarcopenia could be alleviated through adequate nutritional amounts of omega 3, vitamin C, vitamin D, creatinine monohydrate, and antioxidants [51].

Despite the recognized efficacy of exercise programs and nutritional support, these treatment approaches are sometimes unfeasible in sarcopenia patients, especially in frail polymorbid elderly populations [52]. Alternatively, several pharmacological agents have been proposed as therapeutic strategies, even though no drug has been approved for sarcopenia treatment by the US Food and Drug Administration (FDA) or European Medicines Agency (EMA) [42,43,53,54]. Among the pharmaceutical possibilities are included myostatin inhibitors, anabolic or androgenic steroids, growth hormones, angiotensin-converting enzyme (ACE) inhibitors, troponin activators, appetite stimulants, activating II receptor drugs, and β-receptor blockers. Nonetheless, these treatments have variable efficacy and are also associated with side effects. For instance, testosterone administration has been linked with an increased risk of cardiovascular disease and worsened benign prostatic hyperplasia, whereas treatment with growth hormones can lead to fluid retention and orthostatic hypotension [42,54].

Even though few therapeutic strategies can be conventionally employed, experts suggest that it is better to combine the possibilities into a multimodal approach [49]. Moreover, it is generally accepted that the best way to combat sarcopenia is by preventing disease occurrence and development [4].

## 4. Emerging Treatment Approaches

As ongoing research reveals sarcopenia’s intricate molecular mechanisms and therapeutic targets, it becomes clear that novel treatments must be urgently developed. In particular, the lack of clinically-approved pharmaceuticals for this illness intensifies the need for identifying/establishing new drug formulations and diversifying treatment approaches [22,54,55,56]. Taking into account the research directions recognized in recent literature, the following subsections describe the potential of several emerging therapeutic formulations, drug delivery systems, stem cell therapies, and tissue-engineered scaffolds. Moreover, relevant clinical trials on sarcopenia are reviewed in the end.

### 4.1. New Therapeutic Formulations

Several promising therapeutic agents are currently being evaluated by various pre-clinical studies. For example, exerkines like interleukin 6, TNF-α, interleukin 15, fibroblast growth factor 21, irisin, apelin, and others are investigated for their potential to prevent the loss of muscle mass/strength and improve physical performance. Following examination of the outcomes of muscular contraction, particularly during and after exercise, it was discovered that exerkines have autocrine, paracrine, and endocrine effects [52].

Increasing interest has also been invested in investigating growth-promoting agents as solutions to sarcopenia. Myostatin inhibitors, testosterone, and selective androgen receptor modulators (SARMs) have been recognized for their ability to increase lean mass. However, further research is needed to confirm if this translates to increased muscular strength and physical performance in older persons with sarcopenia [23].

As people age, the circulation levels of many anabolic hormones decrease, which may lead to muscle mass and function changes. As a result, hormone modulation has been examined as the foundation of emerging sarcopenia therapeutic strategies, with testosterone supplementation being the most commonly involved drug for enhancing muscle mass and increasing muscle-protein anabolism [12,53,57]. However, the findings of testosterone replacement therapy studies in males vary depending on the participant’s age, pre-treatment testosterone levels, and administration methods. These variables impede the assessment of therapy’s impact on disability and physical performance. In addition, potential side effects (e.g., peripheral edema, gynecomastia, polycythemia, and sleep apnea) have to be considered in parallel with therapeutic advantages. Another important disadvantage related to testosterone is that, in high amounts, this hormone may increase the risk of prostate cancer, imposing careful planning and monitoring during testosterone replacement therapy [53].

As a safer and more appealing approach to long-term testosterone treatment, SARMs have more recently been investigated for combating muscle wasting. SARMs are nonsteroidal ligands that bind to androgen receptors (AR) with tissue-selective androgenic signaling, offering anabolic effects on muscle similar to testosterone but with fewer side effects. These new nonsteroidal compounds also present therapeutic potential for women, being also linked to tissue-selective activity and improved pharmacokinetics [23,50,53]. Significant promise for hormone modulation therapies may also come from dehydroepiandrosterone (can enhance muscle mass and strength in both males and females), tibolone (can influence muscle anabolism acting by binding androgen receptors in muscle fibers and increasing serum-free testosterone, growth hormone, and insulin growth factor 1 (IGF-1) levels), estrogen (can suppress inflammatory cytokines and improve muscle strength in women), growth hormone (can lead to the proliferation of muscle satellite cells and improve muscle function), and ghrelin (can increase the lean mass and physical performance) [53]. Nonetheless, in spite of emerging evidence relating age-related hormonal alterations to the development of sarcopenia, the clinical efficacy of hormone supplementation for the therapy of sarcopenia remains to be more deeply assessed and confirmed in future studies [12].

Given their anabolic properties, branched-chain amino acids (BCAAs) may hold promise for counteracting muscle atrophy in sarcopenia patients. Oral supplementation with BCAAs (i.e., leucine, isoleucine, and valine) can stimulate protein synthesis via the mTOR pathway while inhibiting protein breakdown by decreasing Atrogin-1 and MuRF-1 protein levels and altering the activity of Ub-proteasome. Thus, adequate protein levels are maintained, leading to improved muscle health in sarcopenic conditions [58,59]. The administration of leucine was particularly investigated, and it was revealed to be a well-tolerated BCAA supplement. In elderly individuals, it leads to specific improvements in sarcopenia criteria such as functional performance measured by walking time and enhanced lean mass index [60]. However, such nutritional supplements should be used not only as a single intervention but included in a more complex patient-specific therapeutic plan [59].

Exciting prospects for sarcopenia treatment may also arise from the administration of exercise mimetics (also known as exercise pills), especially for patients dealing with difficulties in physical activity. These mimetics imitate the benefits of exercise without involving physical effort. When tested in adult mice, the combination of GW1516 (i.e., PPARβ/δ agonist) and exercise training was noticed to boost oxidative myofibers and running endurance. Similarly, AICAR, an AMPK agonist, enhances running endurance in sedentary mice. Activating PPARδ and AMPK causes large transcriptional alterations in the metabolic skeletal muscle genome, making them prospective therapeutic targets. Despite being in the pre-clinical stage due to adverse effects, the development of exercise mimetics is critical for patients who are unable to engage in physical activity, such as those on bedrest or suffering from severe sarcopenia, and warrants further research in this field [12,61].

Despite the few new therapeutic possibilities, drug discovery and development are costly and time-consuming, with a low success approval rate for emerging formulations and the necessity for numerous testing stages before clinical implementation. Thus, as an alternative, drug repurposing has been gathering more attention in recent years toward maximizing the effects of pharmacotherapies for sarcopenia [23,55]. Drugs that are currently used for other diseases may be explored in sarcopenia based on their pro-anabolic or anti-inflammatory properties that translate to improvements in skeletal muscle mass and function [23]. For instance, some drugs now utilized for the treatment of type 2 diabetes mellitus may possess mechanisms of action that are applicable to the prevention and therapy of sarcopenia, both in individuals with type 2 diabetes and those without diabetes [62]. One particularly appealing drug examined for sarcopenia prevention is metformin, which may have the ability to delay aging and the incidence of age-related diseases, with its effects on muscle and exact mechanisms of action still requiring further elucidation [12,63]. Up to now, it has been established that metformin inhibits proinflammatory cytokine production and intracellular pathways activated by inflammation, increases circulating levels of irisin, and suppresses cellular senescence in multiple tissues (including skeletal muscle) [62].

Considering the close relationship between skeletal muscle function, glucose, and fat metabolism in both healthy individuals and those with comorbidities, several other drugs, such as BIO101, GLP-1 receptor agonists, DPP4 inhibitors, and SGLT2 inhibitors, stand out as promising options to be evaluated in sarcopenia clinical studies [23,57,62].

Besides the promise of synthetic drugs, increased attention has also been drawn to natural alternatives. Given that sarcopenia is an age-related illness, several natural compounds with anti-aging properties have been considered [12]. In this respect, the potential of ursolic acid and tomatidine has been explored for sarcopenia treatment, remarking that these natural compounds produce numerous small positive and negative changes in mRNA levels in aged skeletal muscle, with remarkably similar mRNA expression signatures, repressing a subset of the mRNAs linked to the regulation of oxidative and other stress responses [12,64]. Ursolic acid has also been rendered effective for improving muscle tissue when combined with resistance and endurance exercise. However, the effects of its intake have some limitations in human tests [50]. For instance, according to the study of Cho and colleagues [65], ursolic acid supplementation in healthy adults did not have a significant impact on muscle mass and strength. In addition, Bang et al. [66] concluded that the combination of strength training and 8 weeks of ursolic acid supplementation enhanced muscle strength but not lean body mass compared with strength training alone. Consequently, further research is needed to fully elucidate ursolic acid supplementation outcomes in humans affected by sarcopenia [50].

Another useful compound is urolithin A, which was reported to be efficient for increasing muscle function and enhancing exercise capacity in rodents, thus showing promise for future application in improving mitochondrial and muscle function in more advanced settings [67]. More recently, urolithin A administration was also investigated in a placebo-controlled trial in middle-aged adults, showing improvements in muscle strength and performance [68]. In addition, a study performed on patients between 65 and 90 years of age revealed that urolithin A supplementation is beneficial for muscle endurance and mitochondrial health [69].

Curcumin is another natural compound with advantageous properties for sarcopenia management [70,71]. Curcumin could benefit muscle health by preserving satellite cell number and function, maintaining the mitochondrial function of muscle cells, suppressing inflammation, and reducing oxidative stress. Through various mechanisms, curcumin contributes to maintaining muscle mass and performance, with numerous studies demonstrating its beneficial properties [72,73]. Combined with a reduction in food intake, curcumin improved skeletal muscle health in aged skeletal muscle [74], while in HFD-fed animals, short-term curcumin therapy considerably reduced reactive oxygen species (ROS) levels in muscles [75]. Curcumin was also reported to prevent muscle damage by regulating the NF-kB and Nrf2 pathways [76], hinder muscular atrophy by altering genes associated with it and boosting antioxidant capacity [77], protect oxidative stress-induced C2C12 myoblasts [78], and diminish CKD-related mitochondrial oxidative damage and dysfunction through preventing GSK-3 activity in skeletal muscle [79]. Despite the great potential of curcumin for sarcopenia treatment, in-depth research is required to establish details related to the delivery route, exact dosage, and safety in humans [72].

Several studies have indicated the potential of vitamin D supplementation in dealing with sarcopenia symptoms. Specifically, vitamin D has reportedly suppressed muscle atrophy and enhanced muscle strength. However, these beneficial effects on muscle tissue are controversial, given that this vitamin’s underlying mechanisms of action are still not fully elucidated. Hence, additional studies are required to confirm the directed correlation between vitamin D supplementation and improved health status in sarcopenia patients [80].

The effects of sarcopenia can also be alleviated by green tea catechins. These compounds were noted to act on skeletal muscle cells, potentially inhibiting muscle mass loss [50]. Additionally, Mafi et al. [81] have demonstrated that the combination of resistance training and epicatechin supplementation has a significant positive influence in improving muscle growth factors and preventing sarcopenia progression.

**Figure 3:**
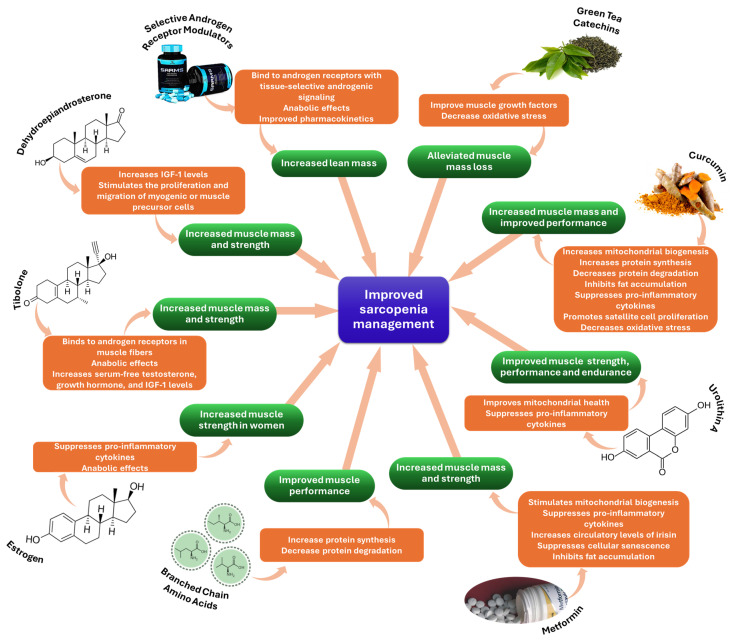
Overview of emerging therapeutic agents for sarcopenia treatment.

For clarity, Figure 3 offers an at-glance perspective on the above-discussed therapeutic strategies for improving sarcopenia management.

### 4.2. Drug Delivery Systems

As age-related muscle loss has no local lesions, therapeutic agents are delivered systemically, risking the exposure of healthy organs (e.g., liver, kidneys) to unwanted side effects. In this context, research is imposed to develop drug delivery systems that can ensure targeted cargo release within muscle tissues. Only a few studies have reported on fabricating delivery systems endowed with targeted motifs for skeletal muscles [16].

One example of a delivery system with muscle-targeting function has been created by Jativa et al. [82]. Specifically, the researchers have developed a generation 5-polyamidoamine dendrimer (G5-PAMAM) functionalized with a skeletal muscle-targeted peptide, ASSLNIA (G5-SMTP) and complexed with a plasmid encoding firefly luciferase. Thus, the authors successfully combined two nanocarrier components, synergistically enabling targeted skeletal muscle cell recognition and improving intracellular gene delivery within skeletal muscle cells. Differently, Gao et al. [83] have demonstrated the muscle-targeting potential of a peptide: CP05. In this regard, the research group has constructed CP05-loaded dystrophin splice–correcting phosphorodiamidate morpholino oligomer (EXOPMO). This delivery system showed enhanced muscle dystrophin expression, improved muscle function, and no detectable toxicity levels.

Interesting alternatives have been envisaged, such as employing nanoparticles for muscle regeneration. For instance, Poussard et al. [84] have investigated the cellular uptake of fluorescently labeled silica nanoparticles by the C2C12 muscle cell line. The authors reported that the design nanoconstructs were uptaken via an energy-dependent process involving macropinocytosis and clathrin-mediated pathway and further clustered in lysosomal structures. Nanoparticle internalization induced an increase in apoptotic myoblasts, enhanced cell fusion, and stimulated the formation of myotubes in a dose-dependent manner.

Research interest was also noted in developing better delivery options for myostatin inhibitors. Ran et al. [85] have anchored myostatin propeptide to the surface of exosomes, fusing it into the second extracellular loop of CD63 (EXOpro). Through repeated administration in mdx mice, EXOpro managed to accelerate muscle regeneration and growth, subsequently enhancing muscle mass and performance. Thus, the designed delivery system is a good candidate for increasing myostatin propeptide’s serum stability and inhibitory efficacy.

Alternatively, Michiue et al. [86] have proposed the delivery of myostatin inhibitory-D-peptide-35 (MID-35) by iontophoresis. This non-invasive transdermal drug delivery strategy uses weak electricity to transport MID-35 from the skin surface to skeletal muscle. Concerning the outcomes, it was observed that skeletal muscle mass increased 1.25 times, the percentage of new and mature muscle fibers tended to enhance, and there were induced alterations in the levels of mRNA of genes downstream of myostatin. Therefore, this delivery possibility represents a valuable therapeutic approach for managing sarcopenia.

Transdermal drug delivery has also been approached for testosterone delivery, yet only modest results have been reported. Specifically, Kenny et al. [87] have investigated transdermal testosterone gel supplementation in a randomized trial. The gel treatment resulted in increased testosterone levels, a positive impact on axial bone mineral density, reduced fat mass, improved lean mass, and no changes in muscle strength or physical performance. Thus, to further improve the effects of testosterone in sarcopenia patients, it is important to find better delivery alternatives. According to the metanalysis elaborated by Skinner et al. [88], intramuscular testosterone replacement therapy offers a 3–5 times increase in muscle mass and strength than transdermally administered testosterone. One more emerging delivery possibility is using microneedle patches loaded with micron-sized needle arrays for non-invasively injecting therapeutic agents to treat musculoskeletal disorders [89]. Nonetheless, further studies dedicated to sarcopenia need to be performed for clarifying the exact outcomes of microneedle patch administration in this disease.

**Figure 4:**
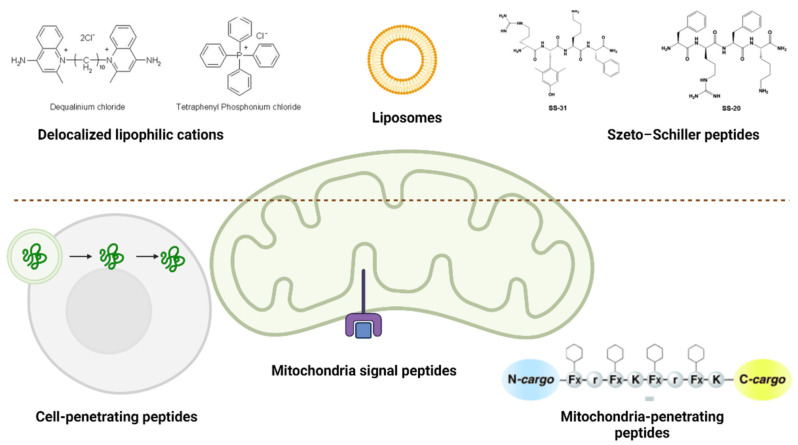
Passive (above the dotted line) and active (below the dotted line) mitochondria delivery strategies. Reprinted from an open-access source [6].

Given the involvement of mitochondria in the pathophysiology of sarcopenia, innovative solutions against this disease can be focused on developing mitochondria-targeting drug delivery systems. The optimal technique for targeting mitochondria is to use 1–1000 nm particles that can directly activate myotubes or inflammatory cells. Two types of targeting can be differentiated: passive and active (Figure 4). Passive targeting depends on the delivery systems’ physical and chemical features, whereas active targeting is related to particular interactions (e.g., ligand-receptor or antigen-antibody) at mitochondrial locations [6].

Compounds can enter mitochondria both actively and passively, acting as scavengers or substitute molecules. However, several of these compounds must be evaluated in vivo for the therapy of sarcopenia. Pre-clinical studies significantly support their potential usefulness in maintaining mitochondrial quality and function, counterbalancing oxidative stress, and reducing mitochondrial death. Creating compounds/delivery vehicles targeting skeletal muscle mitochondria could overcome numerous problems associated with current therapeutics, boosting efficacy while minimizing toxicity [6].

For instance, Pin et al. [90] have recently investigated the possible anti-cachectic benefits of Mitoquinone Q (MitoQ), one of the most commonly utilized mitochondria-targeting antioxidants. The researchers reported that MitoQ administration is an effective method of improving skeletal muscle mass and function in tumor hosts as this formulation could enhance β-oxidation in the muscle tissue, promote a shift in muscle metabolism and fiber composition from glycolytic to oxidative, and decrease myosteatosis.

Differently, Campbell et al. [91] performed in vivo tests on the activity of SS-31 (elamipretide), reporting its advantageous effects. In more detail, treatment with SS-31 improved mitochondrial quality, reversing age-related decline in maximum mitochondrial ATP production and enhancing exercise tolerance without an increase in mitochondrial content. Moreover, SS-31 treatment could restore redox homeostasis in the aged skeletal muscle of mice.

In addition to the above-discussed studies, the advancements made in developing mitochondrial drug delivery systems for other diseases [92] (e.g., cancer [93,94,95,96], Alzheimer’s disease [97,98,99], diabetes mellitus [100,101], ischemia-reperfusion injury [102,103,104]) hold promise for creating nanocarriers of use for sarcopenia treatment as well.

Important advancements noted in exosome-based delivery systems may also aid in developing performant therapeutic formulations for sarcopenia [105], especially as exosomes derived from human skeletal myoblasts or whey protein can improve muscle regeneration [106] and muscle protein synthesis [107]. Furthermore, exosomes have been researched in relation to different diseases, leading to promising results in the treatment of type 2 diabetes [108], gastric cancer [109], sepsis-induced kidney injury [110], myocardial infarction [111], hindlimb ischemia [112], temporomandibular joint osteoarthritis [113], and Parkinson’s disease [114]. Given this advanced scientific context, innovative research strategies in sarcopenia may soon be based on engineered exosomes loaded with specific DNA, RNA, proteins, or drugs to be delivered to targeted muscle cells [105].

### 4.3. Stem Cell Therapies

Even though sarcopenia pathogenesis has not been fully elucidated, there is no doubt that muscle stem cells play an important role [4]. As a result, there is a growing interest in investigating the potential of both muscle-derived and non-muscle-derived cells for skeletal muscle reconstruction, with stem cells emerging as the leading options for repair, especially as mature myocytes have a non-dividing nature [4,115,116].

Several cell types have attracted interest in managing sarcopenia. Myogenic cells, including satellite cells, can be employed to augment the host tissue and produce stable progeny. Pericytes and intervascular cells can develop into muscle fibers and function using paracrine processes. Bone marrow mesenchymal stem cells are the most extensively employed and promising type of non-myogenic cell in regenerative medicine. Mesenchymal stem cells (MSCs) obtained from various sources (e.g., bone marrow, fatty tissue, and the umbilical cord) can enhance stem cell proliferation, angiogenesis, motility, and differentiation via paracrine signals and/or immunomodulation [115,117].

MSCs have been shown in many studies to improve sarcopenia and have been implanted in frail people. MSCs are recognized as valuable therapeutic candidates due to their facile culturing in the laboratory, multipotent differentiating nature, immunomodulatory properties, and ability to secrete various soluble factors. These cells were noted to reduce inflammation and restore cellular function, improving physical performance related to skeletal muscle health. Moreover, MSCs can enhance weak patients’ prognosis by lowering TNF-α levels and inflammation, making them a reliable treatment choice [42,115].

Induced pluripotent stem cells (iPSCs) are also relevant for sarcopenia treatment, as somatic cell reprogramming can be differentiated towards an extensive range of cell types, including skeletal muscle cells [1,118]. iPSCs can be differentiated into myogenic precursors in two ways. The first method uses integrative vectors like lentivirus to overexpress myogenic transcription factors like MyoD and Pax7 in iPSCs. This method is effective; however, vector integration can cause genotoxicity. The other option is to augment iPSCs with myogenic induction factors to simulate embryonic development. Although less efficient, this method is safer and allows differentiated cells to be used therapeutically [1].

Additionally, interesting possibilities are envisaged by studies indicating that transferring mitochondria from stem cells to damaged cells can enhance energy metabolism, preserve mitochondrial function, and enhance overall quality of life. Mitochondrial DNA (mtDNA) mutations contribute to declining mitochondrial function and the onset of sarcopenia-related muscle atrophy. MSCs possess the notable ability to transfer their mitochondria to neighboring cells, aiding in the management of injury and apoptotic stress. This mitochondrial transfer has shown promise in addressing mitochondrial encephalomyopathy in vitro and has opened new avenues for treating sarcopenia linked to mtDNA issues. Since skeletal muscle mitochondrial dysfunction is a key factor in sarcopenia, stem cell transplantation offers a hopeful strategy by providing enhanced cell proliferation, resistance to oxidative stress, apoptosis prevention, and stimulation of mitochondrial biogenesis. Consequently, stem cell-derived mitochondrial transplantation presents a significant potential for sarcopenia treatment, potentially revolutionizing clinical cell therapy practices [115].

On a different note, an innovative study by Wang et al. [119] has revealed the potential of clinical-grade human umbilical cord-derived mesenchymal stem cells (hUC-MSCs) in two models of age-related sarcopenia mice. hUC-MSC transplantation was noted to restore skeletal muscle strength and performance through the cells’ roles in enhancing the expression of extracellular matrix proteins, activating satellite cells, enhancing autophagy, and impeding cellular aging. Therefore, great promise emerges from the use of hUC-MSC-based therapies in treating age-associated muscle diseases.

Despite the many benefits of various stem cell therapies in treating sarcopenia, one main drawback needs to be overcome: transplanting cells alone at the site of injury has limited action, as many cells are known to be lost within a short period of time following transplantation. To address this constraint, tissue engineering has emerged as a promising method that opens up new therapeutic options [1].

### 4.4. Tissue-Engineered Scaffolds

**Figure 5:**
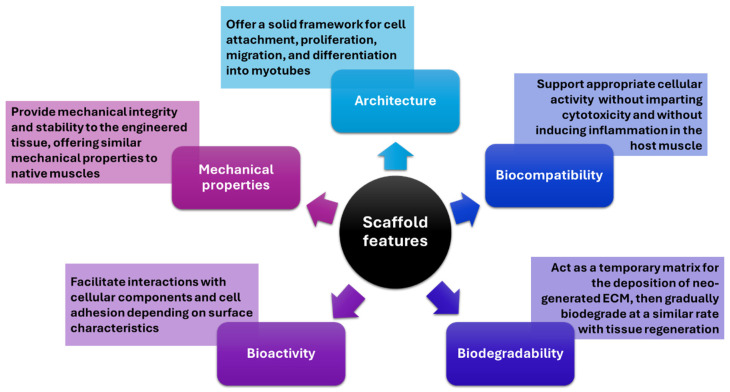
Overview of the main features to be considered when designing a scaffold. Created based on information from [1].

Skeletal muscle tissues have a limited inherent regenerative capacity, requiring external aid when damaged. Tissue engineering emerges as a viable strategy for reconstructing such tissue due to its ability to cultivate cells on a fabricated scaffold (i.e., three-dimensional solid biomaterials) resembling the native extracellular matrix (ECM), thereby facilitating the formation of skeletal muscle tissue. Developing an optimal scaffold is crucial for producing engineered muscle tissue that closely mirrors its natural counterpart and performs analogous functions [1]. In this respect, a series of features have to be considered when designing tissue-engineered scaffolds, as depicted in Figure 5.

One of the essential properties of a scaffold for skeletal muscle tissue engineering (SMTE) is to mimic the structure and morphology of the native tissue. In this regard, the microenvironment plays an important role in the maturation, alignment, orientation, and definition of skeletal muscle tissue. Therefore, when building the scaffold, the guidance of cellular orientation should be considered to efficiently organize muscle cells and obtain a functional construct [1]. Several technologies can be employed to mimic myotube alignment, including the fabrication of parallel linear microchannels [120] and the micro- [121] or nano-patterning of the substrate [122].

Regarding SMTE materials, four classes can be distinguished: natural polymers, synthetic polymers, decellularized scaffolds, and hybrid materials [1]. Natural biomaterials benefit from desirable biocompatibility and biodegradability, making them attractive for developing skeletal muscle tissues in vitro. Moreover, they exhibit adjustable mechanical and morphostructural features, allowing functionalization with moieties of interest (e.g., growth factors, cell adhesion motifs). Among the most employed SMTE materials of natural origin are fibrin, alginate, chitosan, collagen, gelatin, silk fibroin, hyaluronic acid, Matrigel^®^, and decellularized tissue [4,123]. Biomaterials of synthetic origin can also be involved in SMTE. Synthetic polymers, such as polyglycolic acid (PGA), polyethylene glycol (PEG), poly(caprolactone) (PCL), poly(lactic-co-glycolic acid) (PLGA), and poly-l-lactic acid (PLLA), are versatile in use. They can degrade in variable periods (ranging from weeks to years according to the formulation and cross-linking degree), have tunable properties, and are often more cost-effective than natural biomaterials [123].

Besides the potential of biomaterials alone, they can be used in association with cells to generate functional, self-repairing, engineered skeletal muscle. The cell-seeded constructed tissue would benefit from improved vascularization, enhanced innervation, and native muscle-like morphology. Another interesting approach is to combine in situ methods for recruiting host cells and cell delivery methods that activate carried cells. One such method entails the creation of a material capable of attracting satellite cells, activating and inducing their proliferation, and subsequently releasing them into the damaged tissue [124]. An alternative strategy is in vivo SMTE, which entails the introduction of myogenic potential cells into the site of injury via bolus injections or in conjunction with a scaffold biomaterial. Nevertheless, this approach is constrained by the enormous quantity of cells required [123].

On the contrary, the cell-free methodology known as in situ SMTE has been proposed. In this technique, instructive biomaterials are surgically grafted into a muscle defect to stimulate the patient’s endogenous regenerative capacity and promote tissue regeneration through the secretion of bioactive signaling molecules from the implanted biomaterial. Ex vivo SMTE serves as an alternative methodology to in vivo approaches, wherein autologous cells are initially propagated in cell culture and subsequently reintroduced into the defect site for regeneration [123].

Even though these strategies may not specifically target sarcopenia treatment, they provide valuable possibilities that could be adapted for this purpose in the future. As research in tissue engineering and regenerative medicine continues to advance, it is likely that more targeted approaches for managing sarcopenia will emerge.

Moreover, tissue-engineered scaffolds hold great promise as platforms for drug testing. For instance, Rajabian et al. [125] fabricated an in vitro 3D bioengineered senescent adult skeletal muscle (SkM) tissue using primary human myoblasts. The in-lab-created tissue displayed appropriate features for atrophied muscles (e.g., expression of senescent genes, low number of satellite cells, decreased number and size of myofibers, compromised metabolism, altered calcium flux). Compared to young tissue, senescent SkM tissue exhibited a diminished capacity to generate force in response to electrical stimulation and a lack of regeneration capacity in response to injury, which may be attributed to persistent apoptosis and an inability to initiate a proliferation program. Based on these findings, the authors concluded that the designed 3D construct could serve as a platform for drug testing and identifying therapeutic compounds that enhance the function of sarcopenic muscle and a potent model for researching aging [125].

### 4.5. Clinical Trials

**Figure 6:**
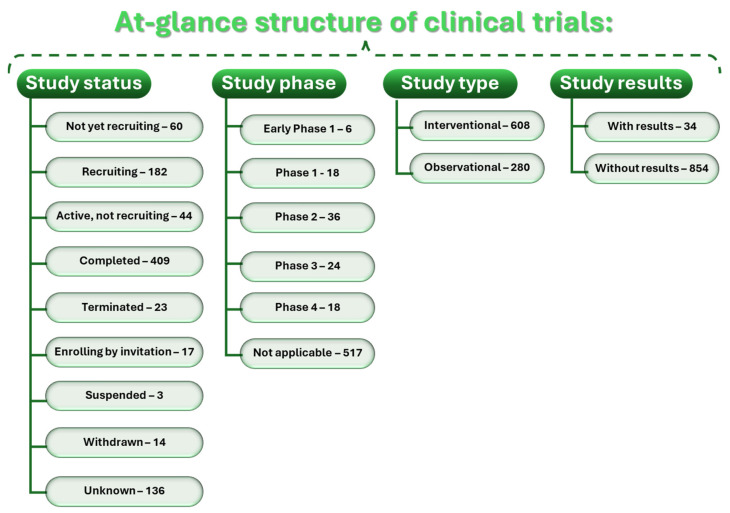
The structure of clinical studies available on ClinicalTrials.gov as of February 2024.

Better-performing therapeutic approaches can also be expected to be provided after completing and interpreting the results of undergoing clinical studies in the field. A total of 891 clinical trials are registered on the ClinicalTrials.gov platform from a search of “sarcopenia”, which are classified as depicted in Figure 6. Restricting the search with “completed”, “interventional”, and “with results” filters led to the list shortening to 29 studies. The retrieved results were further selected manually based on their relevance, and the remaining 24 clinical trials are summarized in Table 1.

A few of the tabulated studies have also been detailed in several publications retrieved from PubMed. For instance, the NCT02333331 study [129] has been described by Rooks et al. [43], revealing bimagrumab’s effects on older adults with sarcopenia. The clinical trial showed no significant difference between bimagrumab and placebo groups in improving physical function. Nonetheless, the tested drug has reportedly increased lean body mass and decreased fat body mass, indicating its potential benefits in managing sarcopenia in association with proper diet and exercise.

As detailed by Lee and colleagues [150], the NCT01989793 clinical study [133] explored losartan’s impact on prefrail older adults. An improvement was noted in molecular and clinical frailty measures with losartan treatment. The study also suggested the potential role of losartan’s non-angiotensin PPARγ pathway in mitigating frailty.

Park et al. [151] analyzed the results of NCT03502941 [134], comparing the anabolic activity of a balanced essential amino acid (EAA) formulation combined with whey protein against a whey protein-based supplement. The combinatorial approach was noted to be highly anabolic, with a dose-dependent response.

As described by Dennis et al. [152], the NCT02261961 study investigated the relationship between immune function, nutritional supplementation, and exercise training outcomes in older adults. The clinical trial revealed the potential benefits of the supplement (containing arginine, glutamine, and HMB) in supporting immune function and muscle growth during resistance training, which are important factors for maintaining/improving muscle mass, strength, and function during aging.

Based on the results obtained throughout the NCT03579693 clinical trial [142], Ahmadi et al. [153] published an article reflecting on the impact of coenzyme Q10 (CoQ10) and nicotinamide riboside (NR) on exercise tolerance and metabolic profile in CKD patients. It was revealed that this treatment strategy improves systemic mitochondrial metabolism and lipid profiles, but exercise VO_2_ peak or total work efficiency remained unaffected.

Lewis et al. [154] and Lyons et al. [155] reported on the findings of clinical trial NCT01869348 [144], which aimed to describe social support patterns of middle-aged and older adults using a mobile app for a behavioral physical activity intervention. The intervention was feasible and acceptable, and participants were willing to use the app’s social network feature to communicate with peers anonymously, most of them actively contributing to the app’s social support network. The effects of the intervention combining wearable physical activity monitors, tablet devices, and telephone counseling were comparable to other wearable activity monitors, indicating potential effectiveness in increasing physical activity and reducing sedentary behavior.

The results of study NCT01874132 [149] have been described by Sousa et al. in two articles [156,157]. Study participants underwent either aerobic training, mixed aerobic and resistance training, or served as controls. Both training programs significantly reduced clinically high triglycerides and total cholesterol, while combined aerobic and resistance training was more effective in chronically modifying lipid profiles. Moreover, adding resistance exercise to aerobic exercise improved factors associated with fall risk.

Several articles have also been published about a few other tabulated clinical trials, yet they did not explore the results concerning sarcopenia. Harris and Dawson-Hughes [158] elaborated on NCT00357214 findings [138], but only discussed the results on insulin sensitivity or glucose control in non-diabetic older adults. In a different approach, Dias et al. [159] discussed trial NCT00104572 [143], focusing on the effects of transdermal testosterone gel or an aromatase inhibitor on prostate volume in older men with no direct correlation with sarcopenia.

Moreover, despite the existence of numerous studies, the enrollment only varies between 11 and 217. Thus, upcoming extensive clinical trials are vital for crafting precision medicine that aligns with the unique characteristics of individual patients, given the variations in causes and clinical presentations among patients [12].

## 5. Conclusions

To summarize, sarcopenia represents a burdening degenerative muscular disease affecting significant numbers of elderly patients. Currently, sarcopenia is mainly managed through nutritional interventions, physical therapy, and lifestyle modification, with no drug yet approved for this disease. As the pathogenesis of sarcopenia is still poorly understood and there is no treatment recognized as universally effective, it is no surprise that research is being undertaken to establish underlying mechanisms of sarcopenia and develop better-performing therapeutic formulations. The main identified research directions for preventing and combating sarcopenia include the administration of various drugs of either synthetic or natural origin, utilization of different delivery methods for enhancing therapeutic activity, employment of stem cells for restoring regenerative potential in damaged tissue, and engineering tissue scaffolds (as a treatment strategy or as platforms for drug testing). However, most of these options have only reached pre-clinical testing, necessitating more rigorous testing before becoming a useful clinical tool.

Adding to the future research studies needed to explore cellular and molecular mechanisms behind sarcopenia, investigations should also focus on improving diagnostic methods. Most patients with sarcopenia are not diagnosed in a timely manner, living with the condition without taking measures against it. Thus, it would be beneficial to focus on identifying biomarkers to aid in early detection of sarcopenia. Moreover, future analyses should also cover investigations related to predicting sarcopenia treatment responses and elaborating personalized and targeted treatment strategies.

To conclude, recent progress has been made in developing advanced treatment methods for sarcopenia, with much promise coming from emerging formulations and ongoing clinical studies. Nonetheless, there is still room for improvement, and supplementary interdisciplinary research is required to reach future breakthroughs.

## Figures and Tables

**Table 1:** Summary of completed interventional studies with results.

ClinicalTrials.gov ID	Official Title	Intervention	Phase	(Estimated) Completion	Enrollment	Ref.
NCT05224453	Comparative Effects of Integrated Physical Training With High Protein Diet Versus Low Protein Diet in COVID-19 Asymptomatic Older Adults With Sarcopenia Symptoms.	Other: Physical training and high protein diet Other: Physical training and low protein diet	N/A	30 November 2021	76	[126]
NCT03809104	Virtual Reality-based Rehabilitation in the Treatment and Prevention of Sarcopenia of Older Residents in Caring Facilities—a Pilot Study in Rural Southern Taiwan	Other: Virtual reality-based rehabilitation programs	N/A	8 October 2019	43	[127]
NCT02628145	Effects of a Resistance Training Program in Older Women With Sarcopenia	Behavioral: Resistance Training Intervention Behavioral: Active Control Group	N/A	July 2016	25	[128]
NCT02333331	A 28 Week, Randomized, Double-blind, Placebo-controlled, Two-part, Multi-center, Parallel Group Dose Range Finding Study to Assess the Effect of Monthly Doses of Bimagrumab 70, 210, and 700 mg on Skeletal Muscle Strength and Function in Older Adults With Sarcopenia (InvestiGAIT)	Drug: bimagrumab Other: placebo	2	28 June 2018	217	[129]
NCT00529659	A Phase IIa Randomized, Placebo-Controlled Clinical Trial to Study the Efficacy and Safety of MK-0773 in Patients With Sarcopenia	Drug: Comparator: MK-0773 Drug: Comparator: Placebo	2	October 2009	170	[130]
NCT02468674	A 24 Week Off Drug Extension, Parallel Group, Study Assessing Durability of Effect on Skeletal Muscle Strength and Function Following a 6-month Double-blind, Placebo Controlled Study Evaluating Bimagrumab in Older Adults With Sarcopenia (InvestiGAIT Extension)	Drug: bimagrumab Drug: Placebo	2	3 December 2018	160	[131]
NCT04830514	Maximizing the Dietary Pattern of Older Adults: the Effects of Protein Intake on Protein Kinetics	Other: Recommended Dietary Allowance of Protein Other: Habitual protein intake Other: Optimal Protein Intake	N/A	15 March 2015	44	[132]
NCT01989793	A Study of Muscle Strength Maintenance in Older Adults	Drug: Losartan Drug: Placebo	2	October 2016	37	[133]
NCT03502941	Effect of an Essential Amino Acid/Protein Composition on Protein Metabolism	Dietary Supplement: 6.3 g of EAAs mixture and whey protein isolate Dietary Supplement: 12.6 g of EAAs mixture and whey protein isolate Dietary Supplement: 12.6 g of whey protein isolate	N/A	17 July 2019	16	[134]
NCT02692235	Carnitine Supplementation and Skeletal Muscle Function in Aging	Dietary Supplement: carnitine Dietary Supplement: placebo	3	July 2017	28	[135]
NCT00957801	Anabolic and Inflammatory Responses to Short-Term Testosterone Administration in Older Men	Drug: Testosterone injection Drug: Testosterone gel Drug: Medrol	4	December 2015	29	[136]
NCT01032733	Biological Effects of Weight Loss Plus Exercise in Obese Older African-American Women: An Investigation of Aging-related Changes in Black and White Women	Behavioral: Lifestyle Counseling Other: Educational Control	2	October 2009	34	[137]
NCT00357214	Effect of Potassium Bicarbonate on Bone and Muscle	Dietary Supplement: Potassium Bicarbonate Dietary Supplement: Sodium Bicarbonate Dietary Supplement: Potassium Chloride Dietary Supplement: placebo (microcrystalline cellulose)	N/A	April 2008	171	[138]
NCT02617511	Omega-3 Fatty Acid Supplementation and Resistance Training on Inflammation and Body Composition in Older Men	Dietary Supplement: Omega-3 Supplementation Dietary Supplement: Placebo	N/A	November 2016	24	[139]
NCT01083901	Acetaminophen and Impaired Musculoskeletal Adaptations to Exercise Training	Behavioral: Resistance training	N/A	March 2011	34	[140]
NCT02261961	Immune Function and Muscle Adaptations to Resistance Exercise in Older Adults	Biological: TDAP Other: Acute Resistance Exercise Other: Resistance Exercise Training Other: Post-training Follow-up Dietary Supplement: Nutritional Supplement (Muscle Armor) Dietary Supplement: Placebo (Kool-Aid)	N/A	30 September 2019	59	[141]
NCT03579693	Cross-over Randomized Controlled Trial of Coenzyme Q10 or Nicotinamide Riboside in Chronic Kidney Disease	Dietary Supplement: CoQ10 Dietary Supplement: Nicotinamide riboside Dietary Supplement: Placebo	2	26 April 2021	26	[142]
NCT00104572	The Effects of Aromatase Inhibition and Testosterone Replacement in Sex Steroids, Pituitary Hormones, Markers of Bone Turnover, Muscle Strength, and Cognition in Older Men	Drug: Androgel (Testosterone Gel) Drug: Anastrozole (Aromatase Inhibitor) Drug: Placebo tablet Drug: Placebo gel Dietary Supplement: Calcium Cardone 500 mg with vitamin D 400 IU	2	January 2015	44	[143]
NCT01869348	IMPACT: Inactivity Monitoring and Physical Activity Controlled Trial	Behavioral: Monitor intervention	N/A	15 April 2016	40	[144]
NCT03119610	The Physiologic Effects of Intranasal Oxytocin on Sarcopenic Obesity	Drug: Oxytocin nasal spray Drug: Placebo nasal spray	1/2	17 December 2019	23	[145]
NCT00475501	5-Alpha Reductase and Anabolic Effects of Testosterone	Drug: Testosterone Enanthate Drug: Finasteride Other: Placebo	2	October 2014	60	[146]
NCT02838979	Randomized Cross-over Trial of Oral L-Glutamine vs. Maltodextrin on Mitochondrial Function in Chronic Kidney Disease	Dietary Supplement: First Intervention (14 days) Other: Washout (3 weeks) Dietary Supplement: Second Intervention (14 days)	2	31 January 2018	11	[147]
NCT02776553	A Physical Activity Program in End-stage Liver Disease: Pilot Study Assessing Changes in Physical Fitness, Sarcopenia, and the Metabolic Profile	Other: Nutritional consultation Behavioral: Physical training program Behavioral: Behavioral modification therapy	N/A	June 2020	20	[148]
NCT01874132	A Randomised Longitudinal Study of Exercise Prescription for Older Adults: Mode and Intensity to Induce the Highest Cardiovascular Health-related Benefits	Behavioral: Exercise training	N/A	September 2014	66	[149]

## Data Availability

Not applicable.
